# Robotic-assisted thoracoscopic left secondary carinal resection and reconstruction with lung preservation for bronchial squamous cell carcinoma

**DOI:** 10.1016/j.xjtc.2025.102191

**Published:** 2025-12-18

**Authors:** Liang Chen, Wenjun Dai, Yufei Zhang, Jianxin Shi, Wenyong Zhou

**Affiliations:** a Department of Thoracic Surgery, Shanghai Chest Hospital, Shanghai Jiao Tong University School of Medicine, Shanghai, China; b Department of Prevention and Healthcare, Jinyang Community Health Service Center, Shanghai, China

**Figure:**
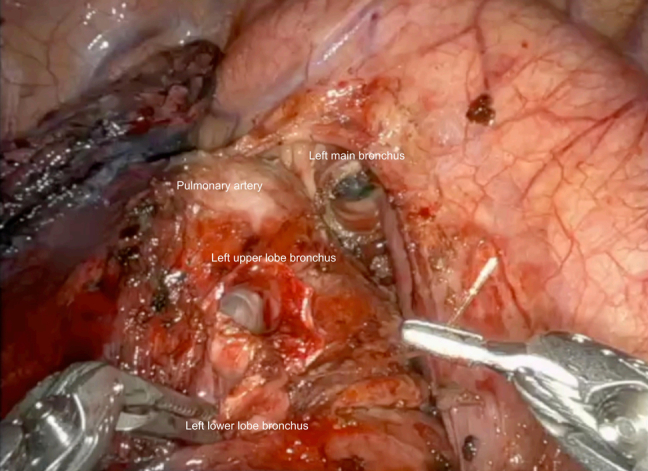
Completed left secondary carina ready for anastomosis to the left main bronchus.

**Central Message**

First robotic-assisted thoracoscopic secondary carinal resection and reconstruction demonstrates the feasibility of complex airway reconstruction through a truly minimally invasive approach.

Secondary carinal resection and reconstruction with lung preservation is a highly complex procedure, performed in carefully selected patients at specialized centers. Its technical challenges mainly arise from anastomotic difficulties, even when performed via an open surgical approach. Limited literature has reported this procedure using a minimally invasive approach.^1^ The advent of Da Vinci robotic-assisted thoracoscopic surgery (RATS), which provides 3-dimensional (3D) visualization of anatomic structures and enhanced maneuverability with wristed instruments, facilitates precise suturing and complex anastomoses.^2^ Here, we report the first case of RATS left secondary carinal resection and reconstruction with lung preservation for bronchial squamous cell carcinoma.

## Case Report

This study was approved by the Ethical Committee of Shanghai Chest Hospital, Shanghai Jiao Tong University School of Medicine (ethical number: KS1992; date: December 21, 2019). Written informed consent was obtained from the patient for publication of this report and any accompanying images. A 63-year-old man presented to Shanghai Chest Hospital with a dry cough. Computed tomography of the chest revealed a lesion in the left main bronchus. Bronchoscopy identified a neoplasm invading the origins of the left upper and lower lobar bronchi, and biopsy confirmed squamous cell carcinoma. Positron emission tomography/computed tomography revealed no lymph node involvement or distant metastasis. After the cardiopulmonary function assessment, the patient was scheduled for RATS left secondary carinal resection and reconstruction.

**Figure 1:**
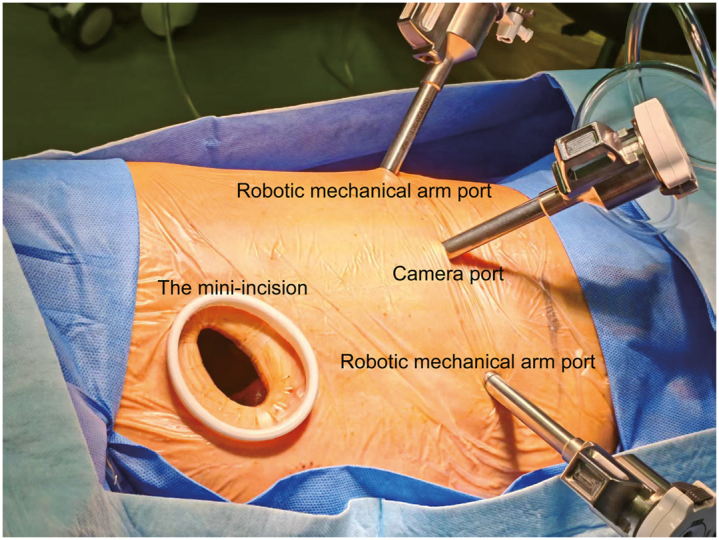
The ports and mini-incision placement.

After intubation with a right-sided double-lumen endotracheal tube for single-lung ventilation, the patient was placed in the right lateral decubitus position. Ports for the camera and 2 robotic mechanical arms were placed at the eighth intercostal space along the midaxillary, the anterior and posterior axillary line, respectively. An additional mini-incision was made at the fourth intercostal space before the anterior arm port (Figure 1). Systematic mediastinal lymph nodes dissection including station 4L, 5, 6, 7, 8, 9, and 10 was performed for accurate pathologic staging and optimal surgical exposure. After we isolated the distal left main bronchus, the origins of the upper and lower lobar bronchi, the left secondary carina was transected. Frozen section analysis confirmed negative margins and excluded lymph nodes metastasis. To reconstruct the left secondary carina, the side-to-side anastomosis between the upper and lower lobar bronchi was performed continuously with knotless sutures using an absorbable unidirectional MONOCRYL 3-0 suture (STRATAFIX Spiral Monocryl Plus Suture; ETHICON SXMP1B427), and then the newly reconstructed secondary carina was anastomosed to the left main bronchus using a continuous PROLENE 3-0 suture (Video 1). Finally, the anastomosis site was carefully examined for air leak and inspected by bronchoscopy. The total operating time was 65 minutes with an estimated blood loss of 100 mL.

The postoperative course was uneventful. The drainage tubes were removed and the patient was discharged on postoperative day 7. The final pathologic diagnosis further confirmed squamous cell carcinoma with negative resection margins and lymph nodes.

## Discussion

Primary tracheobronchial malignancies are rare, with an estimated annual incidence of 0.1 per 100,000 individuals, accounting for approximately 0.2% of all respiratory tract tumors and 0.02% to 0.04% of all malignancies.^3^ For endobronchial lesions, lung-preserving bronchial sleeve resection is considered a safe procedure, offering excellent long-term survival. Secondary carinal resection and reconstruction, as a technically demanding procedure, has been described for lesions located in the distal main bronchus, close to or invading the origins of the lobar bronchi. The complexity is increased on the left side because of the proximity of aortic arch and descending aorta to the hilum, and the position of left pulmonary artery directly above the left main bronchus. The review of left secondary carinal resection and reconstruction in well-selected patients at our center revealed a postoperative complication rate of 13.3%.^4^

With advances in minimally invasive surgery, video-assisted thoracoscopic surgery is increasingly used for localized lesions compared with thoracotomy. However, because of the anatomical complexity, few reports have described secondary carinal resection and reconstruction performed via minimally invasive approaches.^1^^,^^5^ To overcome the limitations of conventional video-assisted thoracoscopic surgery, such as instrument rigidity and the lack of 3-dimensional visualization, RATS was used in this case. This technology provides enhanced dexterity and high-definition 3-dimensional visualization, enabling precise dissection within confined anatomical spaces, which is critical for avoiding injury to adjacent structures during reconstruction. Furthermore, the robotic system's tremor filtration and articulated instruments facilitate meticulous bronchial anastomosis, potentially reducing anastomotic complications and improving functional outcomes. Compared with open surgery, RATS significantly minimizes chest-wall trauma, preserves respiratory muscle function, and accelerates postoperative recovery. These combined advantages support the viability of RATS as an alternative for well-selected patients requiring complex airway reconstruction.

In conclusion, RATS secondary carinal resection and reconstruction with complete lung preservation can be performed in well-selected patients, indicating the feasibility of this highly complex procedure in a minimally invasive setting.

## Conflict of Interest Statement

The authors reported no conflicts of interest.

The *Journal* policy requires editors and reviewers to disclose conflicts of interest and to decline handling or reviewing manuscripts for which they may have a conflict of interest. The editors and reviewers of this article have no conflicts of interest.

## Supplementary Data

Video 1 Robotic-assisted thoracoscopic left secondary carinal resection and reconstruction. Video available at: https://www.jtcvs.org/article/S2666-2507(25)00572-3/fulltext.
