# Supplementary figures and images for: Robotic-assisted thoracoscopic left secondary carinal resection and reconstruction with lung preservation for bronchial squamous cell carcinoma
Source: JTCVS Tech. 2025 Dec 18;36:102191. doi: 10.1016/j.xjtc.2025.102191 (PMC13069532; doi:10.1016/j.xjtc.2025.102191)

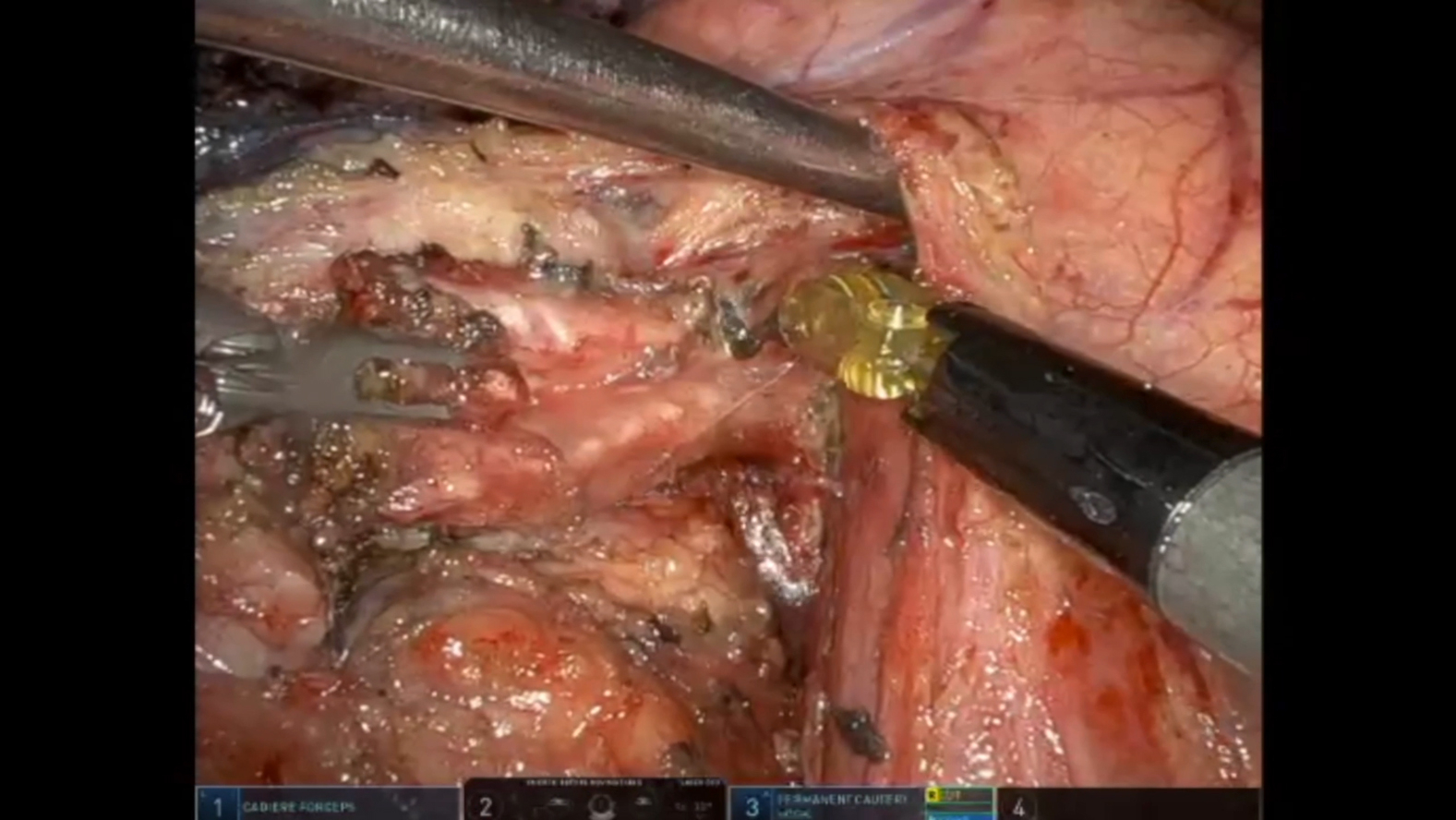

Supplement: Video 1 — Robotic-assisted thoracoscopic left secondary carinal resection and reconstruction. Video available at: https://www.jtcvs.org/article/S2666-2507(25)00572-3/fulltext. [file fx2.jpg]
